# Uloma (Uloma) intricornicula Liu, Ren & Wang, 2007 (Coleoptera, Tenebrionidae, Ulomini): Descriptions of the larva and pupa and new distributional records

**DOI:** 10.3897/BDJ.11.e107036

**Published:** 2023-09-12

**Authors:** Yiping Niu, Guodong Ren, Shanshan Liu

**Affiliations:** 1 Key Laboratory of Zoological Systematics and Application, College of Life Sciences, Institute of Life Science and Green Development, Hebei University, 071002, Baoding, China Key Laboratory of Zoological Systematics and Application, College of Life Sciences, Institute of Life Science and Green Development, Hebei University, 071002 Baoding China

**Keywords:** Uloma (Uloma) intricornicula, immature stages, new distributional records, China

## Abstract

**Background:**

The genus *Uloma* Dejean, 1821 (Coleoptera, Tenebrionidae, Ulomini) comprises more than 200 species and subspecies worldwide, 37 of which are recorded from China. However, the morphology of the immature stages of Chinese *Uloma* have been poorly documented. Up to now, larva and pupa descriptions are available for only one species, Uloma (Uloma) metogana Ren, 2004.

**New information:**

The larva and pupa of Uloma (Uloma) intricornicula Liu, Ren & Wang, 2007, from southern China, are described and illustrated for the first time and are compared with those of U. (U.) metogana Ren, 2004. Differences between male and female pupae of this species are highlighted. New distributional data for U. (U.) intricornicula are also provided. Finally, 13 *Uloma* species from China are formally assigned to the nominated subgenus.

## Introduction

Despite the increasingly recognised importance of improving our knowledge of beetle immature stages, not only for taxonomical and phylogenetical studies, but also for ecological, behavioural and conservation research (Böving and Craighead 1931, Bouchard and Steiner 2004, Giulio and Moore 2004, Beutel and Friedrich 2005, Giulio et al. 2007, Mahlerová et al. 2021), tenebrionid immature stages are still very poorly known (Matthews et al. 2010, Kamiński et al. 2019, Raś et al. 2022).

*Uloma* Dejean, 1821, the most speciose genus in the tribe Ulomini Blanchard, 1845 of Tenebrionidae Latreille, 1802, includes more than 200 described species and subspecies. The genus is globally distributed, except for Antarctica (Schawaller 2000, Schawaller 2015, Merkl and Ando 2018). *Uloma* species are usually found in decaying wood and under the bark, especially in warm and moist forests (Schawaller 1996). Information on the immature stages of *Uloma* is limited to the larvae of only five species and pupae of four species, with most of the original descriptions not detailed enough to permit good morphological comparisons (Perris 1877, Korschefsky 1943, Byzova and Keleinikova 1964, Hayashi 1966, Watt 1974, Skopin 1978, Fontes 1979, Steiner 1995, Klausnitzer 1996, Cherney 2005, Cherney 2006, Liu et al. 2022).

In the present paper, the larva and pupa of Uloma (Uloma) intricornicula Liu, Ren & Wang, 2007 are described in detail on the basis of morphological characters for the first time. Differences between male and female pupae are indicated and the exuvia of the last instar larva is illustrated. U. (U.) intricornicula is the second *Uloma* species from China whose immature stages are described after U. (U.) metogana Ren, 2004 (Liu et al. 2022). Larval and pupal diagnostic characters that make it possible to distinguish the two species are therefore discussed.

**Figure 1.**
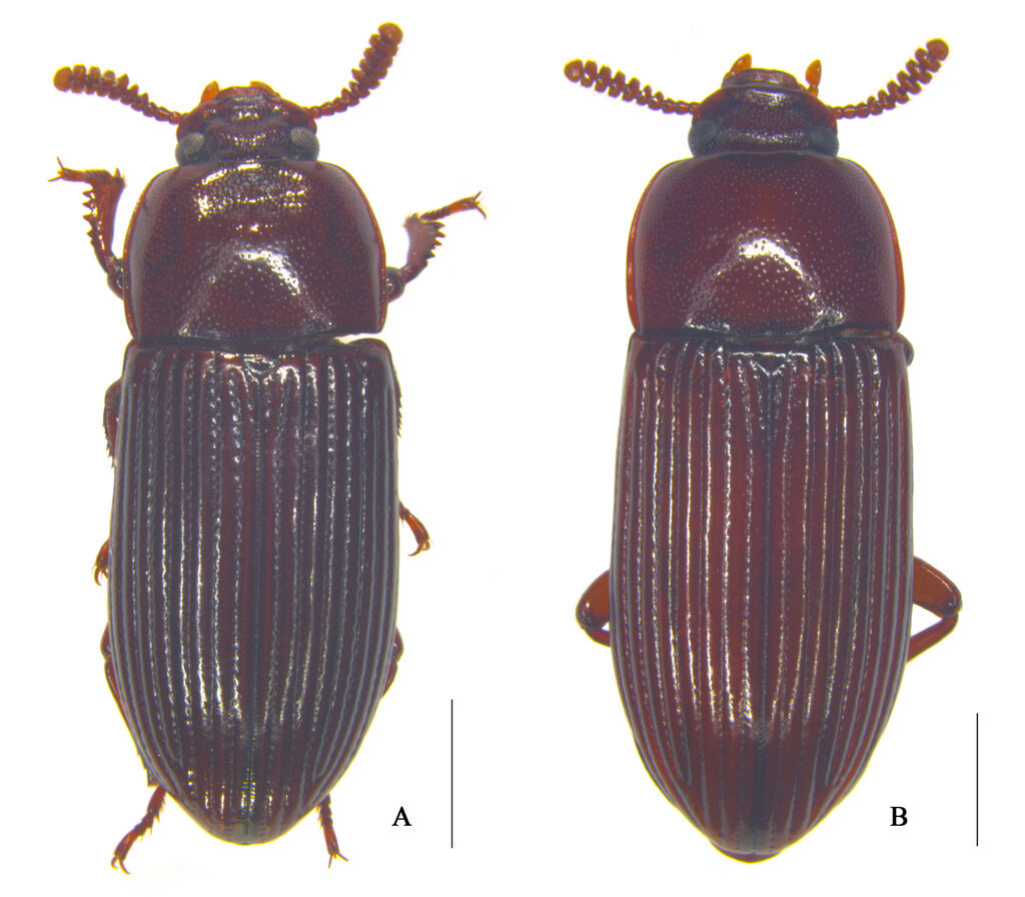
Habitus of the adult of Uloma (Uloma) intricornicula Liu, Ren & Wang, 2007, dorsal view. A. male; B. female. Scale bars: 1 mm.

In addition, the distributional range of U. (U.) intricornicula (Fig. 1) is revised with the addition of new provincial records from Hainan, Guangdong, Sichuan, Chongqing and Guizhou of China, based on the examination of *Uloma* specimens preserved in the Museum of Hebei University.

## Materials and methods

**Figure 2.**
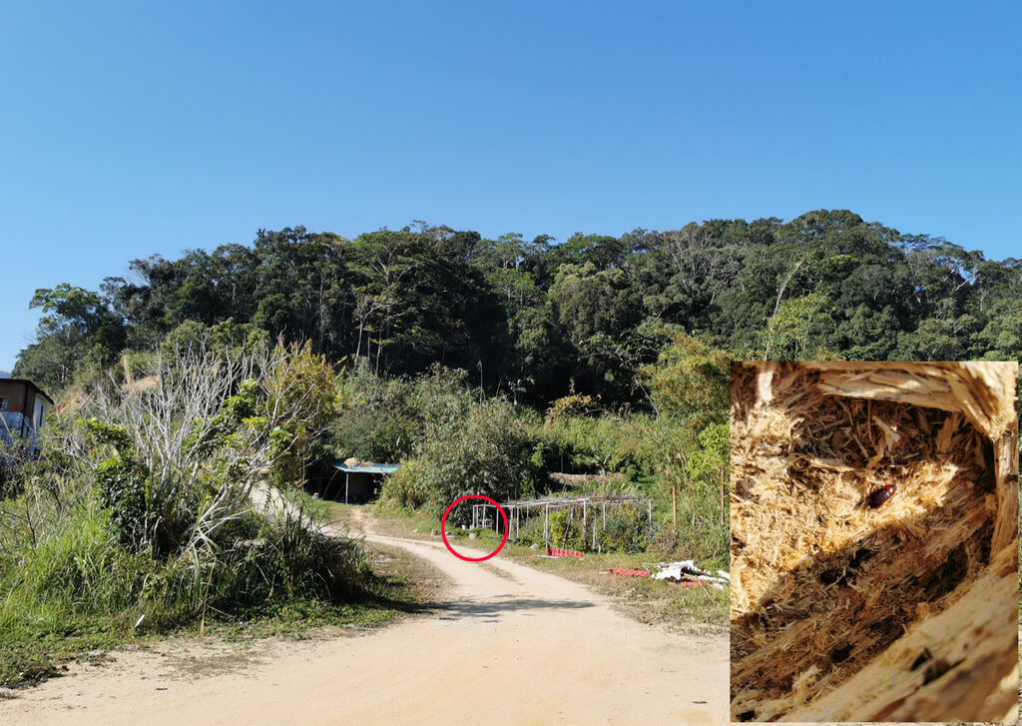
Habitat for Uloma (Uloma) intricornicula Liu, Ren & Wang, 2007. Photo by Caixia Yuan at Jianfengling, Ledong, Hainan, China, on 24 January 2021. The collecting location is outlined in red.

Larvae and pupae of Uloma (Uloma) intricornicula were obtained from eggs laid by eight adult females collected in the field (Fig. 2) by Caixia Yuan on 24 January 2021, from Jianfengling (elev. 799.4 m, 18°44’N, 108°51’E), Ledong Li Autonomous County, Hainan Province, China. In the same place, five adult males were also sampled. Both males and females were collected beneath the bark of rotten trunks. Adults and some soft parts of rotten trunks brought back from the wild were transported to the laboratory and placed in a rectangular semi-transparent plastic box (length: 290 mm, width: 190 mm, height: 140 mm). The plastic box was put into an illumination incubator. Female adults laid eggs in the incubator. Incubator parameters were set as follows: 90% relative humidity (RH), temperature between 24℃ and 25℃, 14.4 h of light and 9.6 h of dark daily periods (intensity of illumination 60%).

Five larvae and four pupae obtained during rearing were fixed in 75% alcohol for the description. Other larvae and pupae were reared to the adult stage.

Other examined adults included: 2 males, 1 female, Mt. Nankun, Huizhou, Guangdong, China, 27 August 2010, Haoyu Liu lgt.; 5 males, 5 females, Changning (elev. 310 m), Sichuan, China, 9 July 2008, Aimin Shi and Guang Lai lgt.; 3 males, 2 females, Mt. Jin Yun, Beibei, Chongqing, China, 7 May 2014, Jianyue Qiu lgt.; 2 males, 9 females, Maolan, Libo, Guizhou, China, 3 August 2010, Yiping Niu and Yong Zhou lgt.; 4 males, 1 female, Mayanghe, Yanhe, Tongren, Guizhou, China, 5–12 June 2007, Fengyan Wang lgt.

All specimens are deposited in the collection of the Museum of Hebei University, Baoding, China.

The larvae and pupae were observed and described using a Nikon SMZ800 stereomicroscope. Photographs were taken with a Leica M205A stereomicroscope, equipped with a Leica DFC450 digital camera and a drawing tube. The morphological terminology of larval and pupal structures follows Steiner (1995), Bouchard and Steiner (2004) and Matthews et al. (2010). More than one larva was examined, so the range of values was given, as were the pupae.

## Taxon treatments

### Uloma (Uloma) intricornicula

Liu, Ren & Wang, 2007

F7630765-E889-59BA-861E-816D0E35EB0B

Uloma intricornicula Liu, Ren & Wang, 2007 - Liu et al. (2007): 71 (type locality: China, Fujian, Guadun).

#### Description

**Figure 3.**
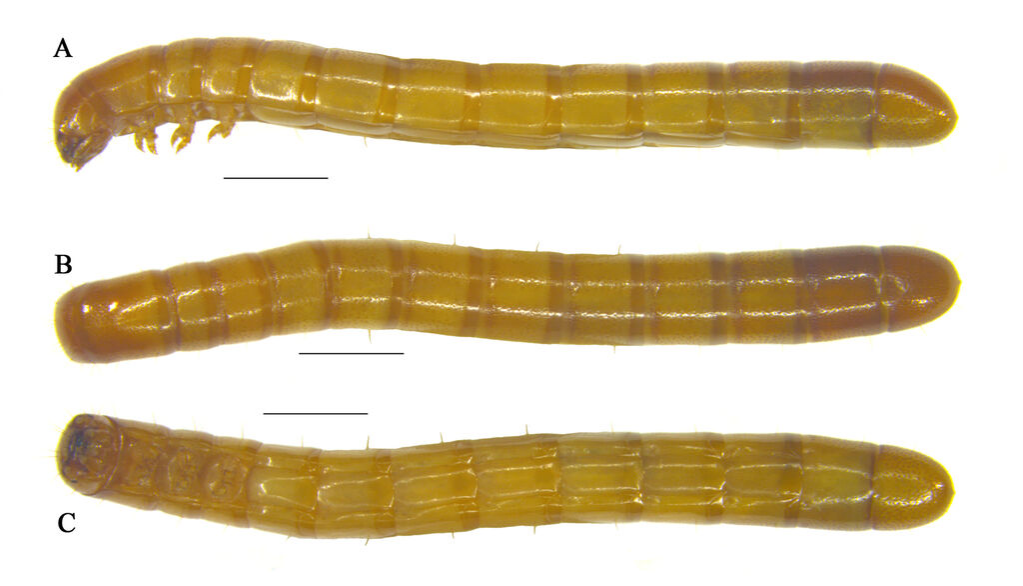
Habitus of the larva of Uloma (Uloma) intricornicula Liu, Ren & Wang, 2007. A. lateral view; B. dorsal view; C. ventral view. Scale bars: 1 mm.

**Larva**: Oligopod (Fig. 3). **Body.** Length 13.5–14.0 mm. Body subcylindrical, ventrally flat and with sharp tail-end; evenly sclerotised both dorsally and ventrally; yellowish-brown, dorsum and both ends brown. Vestiture smooth, suffused with large and round punctures both dorsally and laterally.

**Figure 4.**
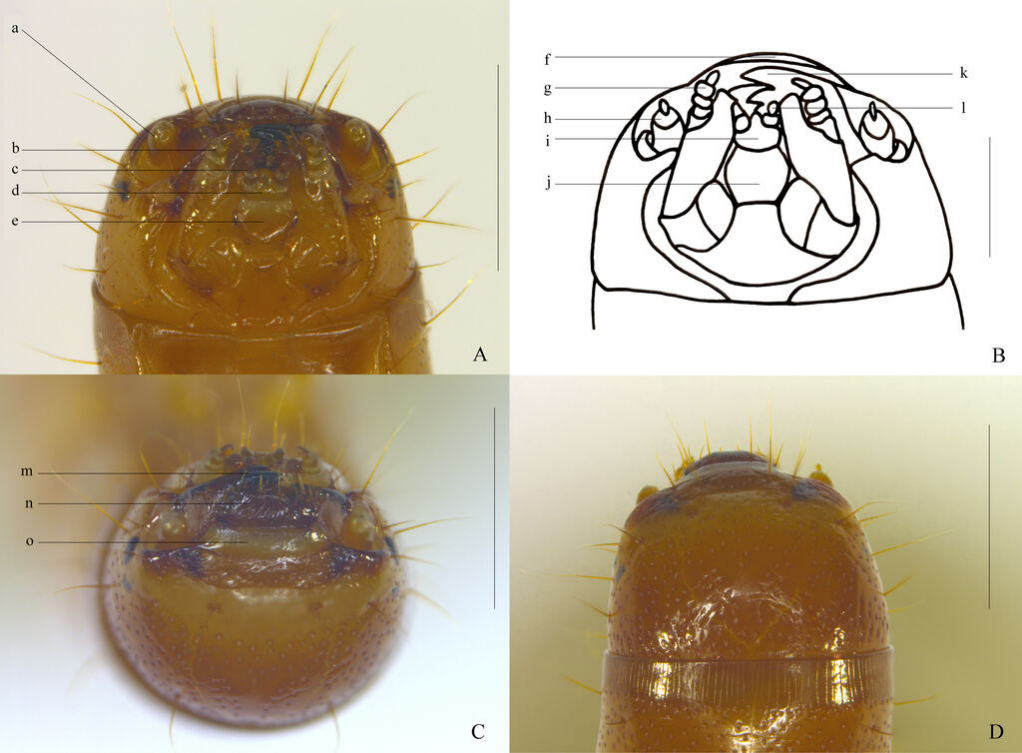
Larva of Uloma (Uloma) intricornicula Liu, Ren & Wang, 2007. A. head, ventral view; B. head, ventral view; C. head, anterior view; D. head, dorsal view. a, h. antenna; b, g. maxillary palpus; c, l. labial palpus; d, i. ligula; e, j. mentum; k, m. mandible; f, n. labrum; o. clypeus. Scale bars: 0.5 mm.

**Head.** Head (Fig. 4) about as wide as prothorax, trapezoidal, slightly convex dorsally; with 10 long setae near the anterior margin, 6 in the middle and 10 near the posterior margin. Frontoclypeal suture evident, almost straight in the middle. Frons slightly convex, frontal sutures broadly V-shaped, distinctly incurved near the centre; median suture barely visible in dorsal view. Clypeus (Fig. 4C) slightly flat, with anterior margin linear, bearing four erect clypeal setae, with the central two longer. Labrum (Fig. 4C) transverse, semi-elliptic, slightly convex, with several short setae on anterior margin and four long erect median setae. Ocelli black, divided into two parts, the upper of which smaller than the lower. Antennae (Fig. 4A, Fig. 4B) shorter than half length of head; antennomere I short; II cylindrical, more than twice as wide as long and three times longer than I, sensorium nearly C-shaped; III thin and short, about 1/6 as long as II and with one long erect median seta and three very short setae around the base of long one at apex of III. Mandibles (Fig. 4B, Fig. 4C) well developed, distally black, extended anteriorly; tridentate apically, with the apical tooth markedly larger than the dorsal and ventral ones. Maxillae almost parallel-sided, with dense setae on the apical inner margin; maxillary palpi (Fig. 4A, Fig. 4B) distolateral, subcylindrical, gradually narrowing towards the apex, with the terminal palpomere slender and short. Labial palpi (Fig. 4A, Fig. 4B) 2-segmented, short. Ligula (Fig. 4A, Fig. 4B) slightly convex, with two long erect setae, anterior margin distinctly protruding in the middle part. Mentum (Fig. 4A, Fig. 4B) subhexagonal, widest in the middle, anterior margin weakly emarginate, with two long erect setae on both sides and posterior part respectively, the latter much thicker and longer.

**Figure 5.**
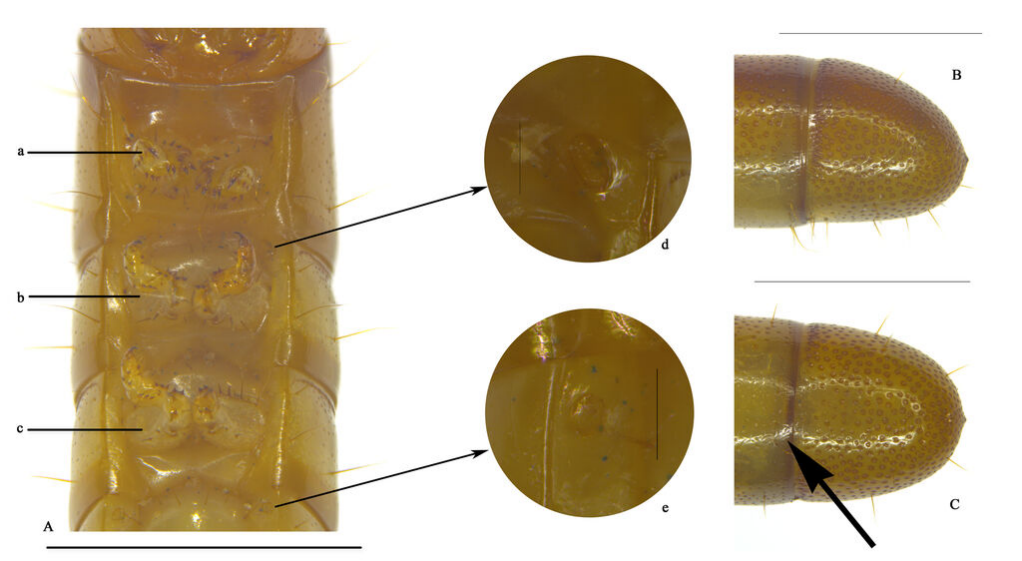
Larva of Uloma (Uloma) intricornicula Liu, Ren & Wang, 2007. A. thorax segments, ventral view; a. prothoracic leg; b. mesothoracic leg; c. metathoracic leg; d. mesothoracic spiracle; e. abdominal spiracle. B. abdominal segment IX, lateral view. C. abdominal segment IX, ventral view. Scale bars: 0.1 mm (d, e), 1 mm (others). The arrow indicates that the anterior margin of the ventral surface of abdominal segment IX is almost straight.

**Thorax.** Thorax 3-segmented. Each thoracic tergum with long erect setae near sides of anterior and posterior margins, distributed as follows: 4 and 3 setae on anterior and posterior margins of prothoracic tergum, respectively; 1 and 2 setae on mesothoracic tergum; and 1 and 3 setae on metathoracic tergum. Prothoracic tergum longer than wide, nearly rectangular in dorsal view, about twice as long as the meso- or metathoracic tergum; ratios between thoracic terga as follows: 1.5: 0.7: 0.9. Meso- and metathoracic terga transverse, nearly rectangular in dorsal view. Mesothoracic spiracles (Fig. 5A) placed on anterolateral margins of tergum, near distinctly separate coxal cavity, visible in ventral view, slenderly oval in shape, large and approximately three times larger than the abdominal spiracles. Prothorax and metathorax without spiracles.

**Legs.** Pro-, meso- and metathoracic legs short, subequal in length and similar in shape (Fig. 5A). Coxa of prothoracic leg thick, longer than other segments, with 5–6 long spiniform setae on anterior margin and 2 on posterior margin; trochanter subtriangular, with 2 short spiniform setae on anterior margin, one on posterior margin and one long hair in the middle; femur subequal in length and width, with sparse short hairs, with 3 short spiniform setae on anterior margin and 5 on posterior margin and one long hair in the middle; tibia much more slender and shorter, with sparse short hairs, with 2 short spiniform setae on anterior margin and 5 on posterior margin; tarsungulus falciform, with 2 thinner short spiniform setae under it. Coxa of mesothoracic leg with 6–7 long spiniform setae on anterior margin and 2 on posterior margin; trochanter with 2 short spiniform setae on anterior margin and one on posterior margin; femur with 3 short spiniform setae on anterior margin and 5 on posterior margin; tibia with 2 short spiniform setae on anterior margin and 4 on posterior margin; others similar to those of prothoracic leg. Coxa of metathoracic leg with 5–6 long spiniform setae on anterior margin and 2 on posterior margin; trochanter with 2 short spiniform setae on anterior margin and only one on posterior margin; femur with 3 short spiniform setae on anterior margin and 5 on posterior margin; tibia with 2 short spiniform setae on anterior margin and 4 on posterior margin; others similar to those of prothoracic leg.

**Abdomen.** Abdomen 9-segmented, gradually and slightly darker towards apex, slightly enlarged backwards. Segments I–IX with denser punctures on the basal dorsal surface in comparison with other parts of abdominal segments. Tergites I–II wider than long, nearly rectangular in dorsal view; III–VIII subquadrate in dorsal view. Tergites I–VII with an arcuate shallow impression respectively near posterolateral margins, with 4 long erect setae on sides of posterior margins; only tergite I with 2 long erect setae on sides of anterior margin. Sternites I–VII nearly rectangular, longer than wide and with a long erect seta near each corner; sternites I with other 4 long erect setae near the anterior margin. Segment VIII without pleural sutures, with 2 long erect setae respectively on posterolateral margins, with 2 erect setae near the anterior margin and 6 near the middle of posterior margin of ventral surface. Segment IX (Fig. 5B, Fig. 5C) with dense even large punctures and sparse long erect setae; dorsal surface with 2 setae; ventral surface with 6 setae at the centre, anterior margin almost straight; tip-end with 4 setae surrounding it. Segment IX parabolic, as long as wide, subcircular in cross-section, tip-end slightly round with a small papilla, but without urogomphi. Anus concealed in posterior part of abdominal tergite VIII, without anal tubes. Abdominal spiracles (Fig. 5A) round, of the same size, opening on anterolateral margins of segments I–VIII.

**Remarks**

The description above is based on the last instar larvae, whose exuvia was preserved in 75% alcohol (Fig. 7F).

The larvae of *Uloma* differ from other known tenebrionine larvae by the presence of an elongate anterior extension on the hypopharygeal sclerome (as in Alleculinae Laporte, 1840), a paraboloid abdominal segment IX with an apical point, the lack of cerci, the lack of pleurosternal sutures on abdominal segment VIII, a reduced anal region and the lack of anal tubes (Hayashi 1966, Watt 1974). Although there is a substantial morphological homogeneity amongst larvae of different species in the genus *Uloma*, we found some important differences between U. (U.) intricornicula and U. (U.) metogana. Namely, mentum is relatively slender and widest in the middle in U. (U.) intricornicula, while it has the maximum width more anteriorly (4/5 from the base) in U. (U.) metogana; protibia with 2 short spiniform setae on the anterior margin in U. (U.) intricornicula, with only one seta in U. (U.) metogana; mesotibia with 2 short spiniform setae on the anterior margin in U. (U.) intricornicula, with only one seta in U. (U.) metogana; abdominal segment IX with slightly round tip-end and the anterior margin of the ventral surface almost straight in U. (U.) intricornicula (Fig. 5C), abdominal segment IX with slightly pointed tip-end and the anterior margin of the ventral surface emarginate in U. (U.) metogana.

However, since available descriptions of other *Uloma* species are not detailed enough to permit good morphological comparisons, it is difficult to distinguish the larvae of U. (U.) intricornicula and U. (U.) metogana from other *Uloma* larvae without direct examination of the specimens.

**Figure 6.**
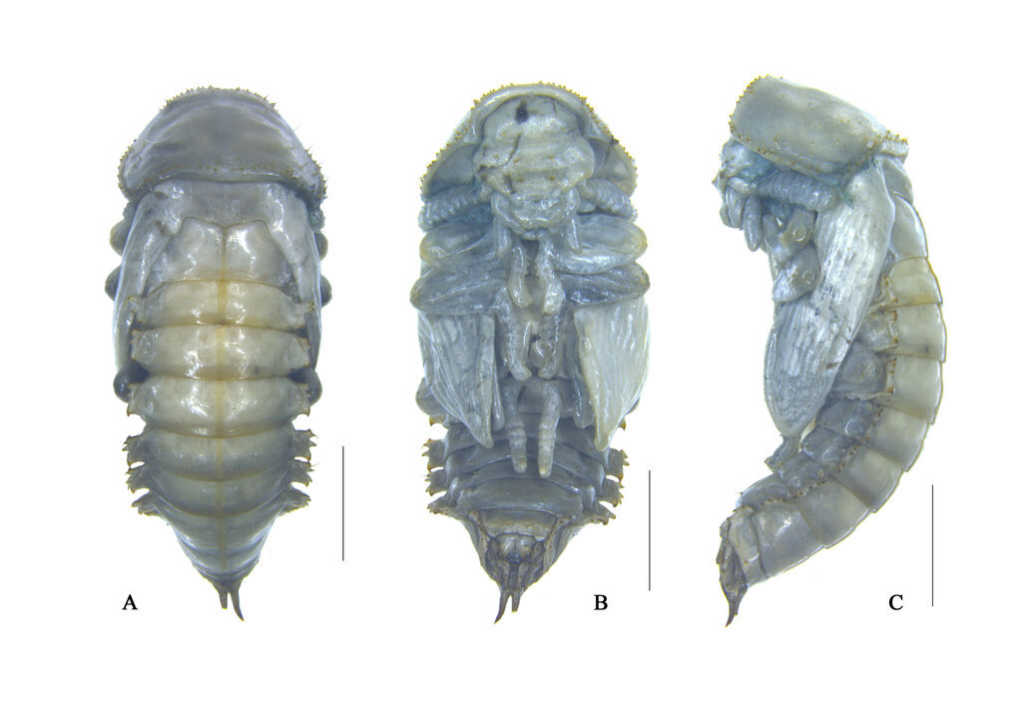
Habitus of the pupa of Uloma (Uloma) intricornicula Liu, Ren & Wang, 2007. A. dorsal view; B. ventral view; C. lateral view. Scale bars: 1 mm.

**Pupa**: Exarate (Fig. 6). **Body.** Length 6.0–7.0 mm, width 2.9–3.1 mm. Body small, slightly elliptic, extremely arched dorsally; grey to light brownish, with darker mouthparts, legs and body backend; most characteristics similar to that of adults of the same species.

**Figure 7.**
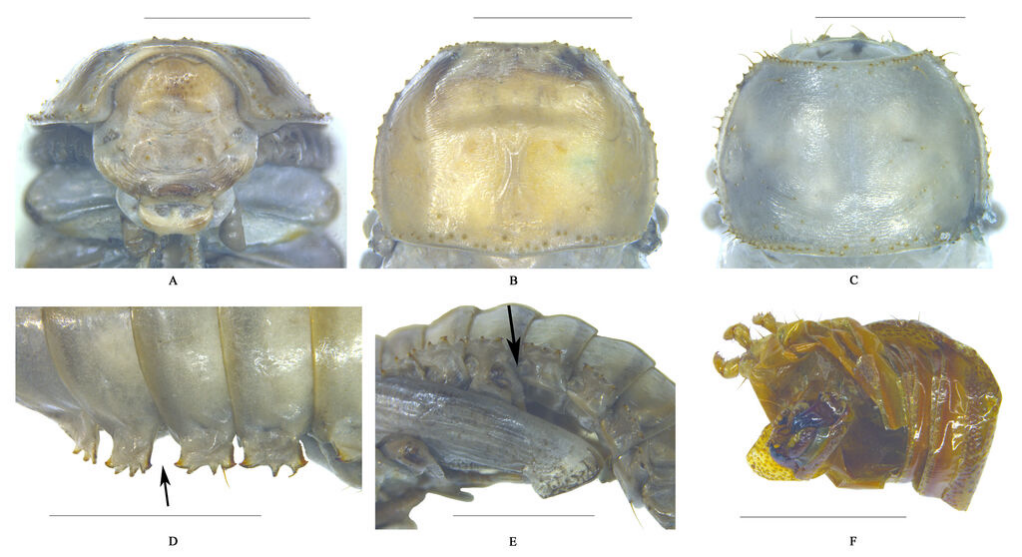
Pupa and exuvia of Uloma (Uloma) intricornicula Liu, Ren & Wang, 2007. A. head, anterior view; B. pronotum, male, dorsal view; C. pronotum, female, dorsal view; D. lateral process of abdominal tergites II–V, dorsal view; E. abdominal tergites I–VII, lateral view; F. exuvia of last instar larvae, lateral view. Scale bars: 1 mm. The arrow (D) indicates the gin trap between segments III and IV. The arrow (E) indicates one of the abdominal spiracles.

**Head.** Visible in ventral view (Fig. 6B, Fig. 7A). Smooth, with transverse wrinkles. Frons depressed, anterior margin with 4 tubercles bearing short erect apical setae in an arc sparsely and posterior margin with 2 far apart tubercles bearing short erect apical setae. Frontoclypeal suture almost linear. Clypeus slightly linear on anterior margin, elevated with 2 small ridges at centre and with a tubercle bearing short erect setae separately on apices of 2 ridges and anterolateral clypeus. Labrum relatively broad, semi-elliptic, anterior margin emarginate at centre, with sparse short hairs. Mandible with sparse long hairs laterally, apices darker, apical tooth distinctly larger than dorsal and ventral ones. Maxillary palpi and labial palpi clearly visible, with sparse long hairs laterally. Eye ovate, with sparse short hairs around it, one of them on anterior margin relatively longer. Antenna thick, claviform, gradually widening towards apex, antennomeres VII–X of distinctly increasing width, XI semi-spherical, V and VII slightly sharply protruding on inner border, I–XI with sparse tubercles bearing short hairs on each apical side and lateral side.

**Thorax.** Thorax 3-segmented. Pronotum slightly transverse, similar in shape to that of adults, about 1.35 times as wide as long, widest near the middle. Pronotum with distinct transverse wrinkles and sparse apical setose tubercles (Fig. 7B, Fig. 7C), tubercles denser on anterior and posterior margins, but sparse on lateral margins and even sparser on disc. Disc slightly convex, with a transverse deep anterior impression. Anterior margin slightly emarginate at centre. Lateral margins markedly arcuate, narrowing forward and less so from widest point to base. Posterior margin slightly protruding at centre. Anterior angles nearly rectangular, posterior angles obtuse. Meso- and metanotum (Fig. 6A) glabrous with irregular wrinkles, distinctly narrower than pronotum, mesonotum slightly wider than abdominal tergite I and metanotum distinctly wider than abdominal tergite I. Mesonotum elevated at centre. Metanotum slightly elevated. Elytral and hind wing sheaths (Fig. 6B, Fig. 6C) glabrous, with several transverse wrinkles; elytra relatively distinctly punctatostriate, but faintly punctate.

**Legs.** Legs similar in shape to that of adults (Fig. 6B). Femur thick, with sparse apical setose tubercles on edges. Tibia almost glabrous, with 2 apical spurs on inner edge; protibia gradually explanate towards apex significantly, meso- and metatibia more slightly explanate. Tarsus relatively slender, with small tubercles on ventral surface. Tarsal claws with 2 small tubercles at apex of ventral surface.

**Figure 8.**
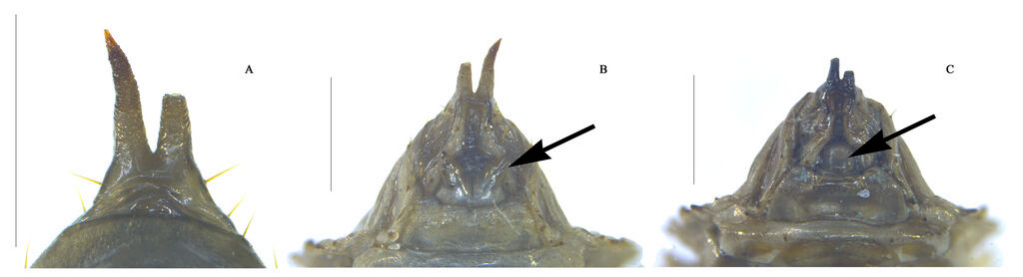
Pupa of Uloma (Uloma) intricornicula Liu, Ren & Wang, 2007. A. urogomphi, male, dorsal view; B. urogomphi, female, ventral view; C. urogomphi, male, ventral view. Scale bars: 0.5 mm. The arrow (B) indicates a pair of tapered styluses of sternite VIII in female. The arrow (C) indicates no tapered styluses in male.

**Abdomen.** Abdomen 9-segmented, dorsomeson quite distinct. Abdominal tergites glabrous, relatively broad, slightly convex and with dense longitudinal wrinkles. Tergites I–VI (Fig. 6C) of similar form, nearly rectangular in dorsal view, length of tergites I–VI distinctly shorter than VII. Tergite VII almost linear on anterior margin, markedly convex in circular arc on posterior margin. Tergites I–VII with well developed rake-like lateral processes. Lateral processes with darker sclerotised edges and each lateral process with several conical projections bearing a short erect apical seta (Fig. 7D). Number of projections from tergites I to VII as follows: 3, 4, 4–5, 4–5, 4–5, 4–5, 2–4. First two projections of tergites II–VI slightly separate. Opposing single projection on lateral processes of adjacent abdominal segments forming anterior and posterior curved teeth of gin-trap structures. Gin trap between segments III and IV as in Fig. 7D. Tergite VIII very short and narrow, about half as long as VII, with sparse and long apical setose tubercles on posterior margin; lateral processes less developed, with 2 or 3 apical setose spines. Tergite IX nearly trapezoid, deeply depressed at centre, with a pair of slender subtapered divergent urogomphi at the end and with sparse small tubercles bearing long erect apical setae on lateral margins and ventral surface. Urogomphi (Fig. 8A) with dense annular wrinkles, gradually slightly narrowing towards apex, directed posteriorly and reflexed finely. Abdominal sternites I–VIII relatively smooth, with fine longitudinal wrinkles and with sparse tubercles bearing long erect apical setae near posterior margin. Sternite VII distinctly and deeply depressed at centre. Abdominal spiracles (Fig. 7E) approximately round, slightly convex, visible on anterolateral margins of abdominal sternites I–VII. Spiracles on abdominal segment I slightly enlarged and hidden beneath wing sheaths.

**Remarks**

The *Uloma* pupae are comparable to adults of the same species in body form, length and most characteristics.

The pupae of U. (U.) intricornicula can be distinguished from those of U. (U.) metogana by the following characters: body small, length 6.0–7.0 mm (larger, length 10.5–11.0 mm, in U. (U.) metogana); pronotum without anterior impression in female (with a shallow one in U. (U.) metogana); first two projections of lateral processes of tergites II–VI slightly separate (distinctly separate in U. (U.) metogana); sternite VII distinctly and deeply depressed at the centre (hardly depressed in U. (U.) metogana).

In addition, we observed some differences between male and female pupae of U. (U.) intricornicula. Sternite VIII with a pair of tapered styluses pointing to the rear in female (Fig. 8B), without in male (Fig. 8C). Pronotum with a transverse deep anterior impression in male, without in female. Antennomeres V and VII slightly protruding at the inner border in male, not protruding in female.

Like in other known species of *Uloma*, U. (U.) intricornicula has the apices of urogomphi spined. The truncated urogomphi apices observed in the examined pupae of U. (U.) metagana might be the result of damage. The apices of urogomphi are very fragile and can break off if shaken slightly. Thus, we suppose that the truncated urogomphi apices in U. (U.) metagana is an artifact and that urogomphi are spined also in this species.

#### Distribution

China: Fujian (Liu et al. 2007), Guangxi (Liu and Ren 2007), Hainan (new record), Guangdong (new record), Sichuan (new record), Chongqing (new record), Guizhou (new record).

#### Notes

##### Remarks

**Figure 9.**
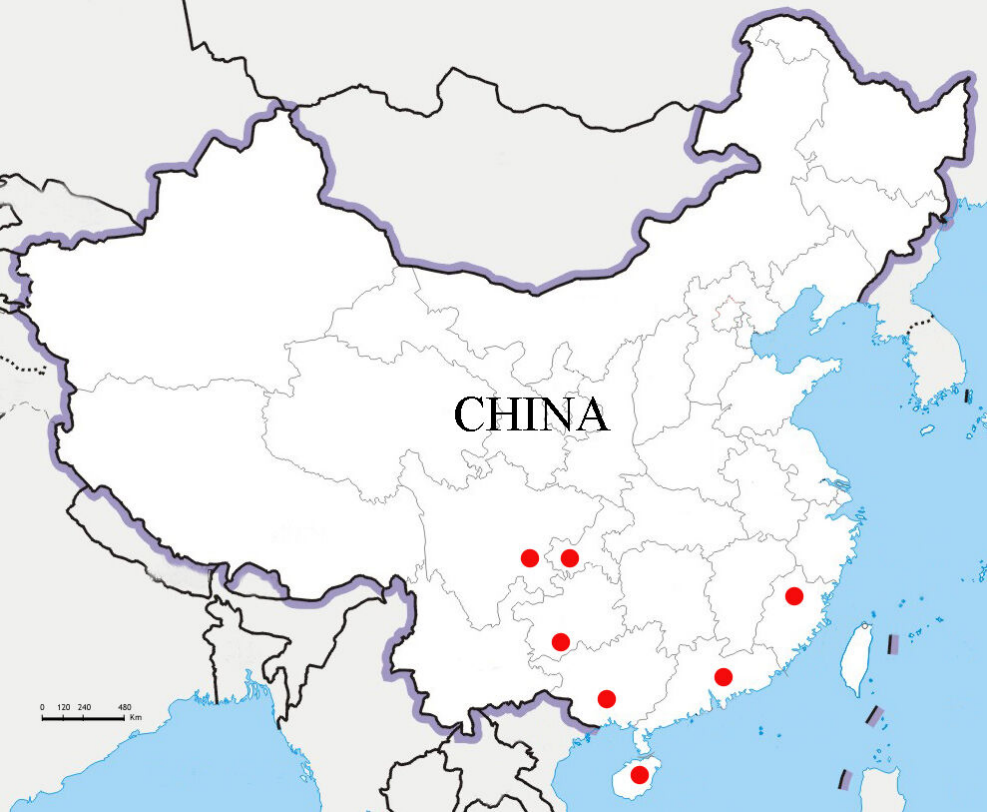
Distribution map of Uloma (Uloma) intricornicula Liu, Ren & Wang, 2007 (red circle). The original map from National Platform for Common Geospatial Information Services.

Uloma (Uloma) intricornicula was described by Liu et al. (2007) from Fujian Province of China. Later this species was mentioned to occur in Guangxi Zhuang Autonomous Region (Liu and Ren 2007). While examining the *Uloma* specimens in the collections of the Museum of Hebei University, we found that U. (U.) intricornicula was also collected from Chongqing Municipality and from Hainan, Guangdong, Sichuan and Guizhou Provinces of China (Fig. 9). Thus, the species seem to be widely distributed in south-western China, where warm-temperate broad-leaved evergreen forests and tropical rain forests occur (Ni 2001).

## Discussion

The genus *Uloma* is currently divided into two subgenera: *Uloma* s. str. and *Apterulomoides*, established by Kaszab (1982), based on Uloma (Apterulomoides) rotundipennis Kaszab, 1982 from Australia. However, the subgeneric status of several described *Uloma* species, including 13 Chinese species, has never been mentioned.

In the present study, we examined type specimens of these species from China and we found that their morphological characters correspond to those of the subgenus Uloma s. str. (metathorax at least half of mesothorax in length, elytra nearly parallel-sided or slightly oval etc.). Therefore, the following Chinese species should be included in the nominate subgenus Uloma: U. (U.) acrodonta Liu & Ren, 2016, U. (U.) compressa Liu & Ren, 2008, U. (U.) contortimargina Liu & Ren, 2007, U. (U.) fengyangensis Liu & Ren, 2016, U. (U.) hirticornis Kaszab, 1980, U. (U.) integrimargina Liu & Ren, 2007, U. (U.) intricornicula Liu, Ren & Wang, 2007, U. (U.) longolineata Liu & Ren, 2007, U. (U.) minuta Liu, Ren & Wang, 2007, U. (U.) quadratithoraca Liu & Ren, 2008, U. (U.) reticulata Liu, Ren & Wang, 2007, U. (U.) valgipes Liu & Ren, 2013 and U. (U.) zhengi Liu & Ren, 2007.

In general, information on the immature stages of *Uloma* is very limited. The description of the larva and pupa of U. (U.) intricornicula provided in this paper revealed that, despite a substantial morphological similarity amongst immature *Uloma*, there are subtle, but clear differences between this species and the recently-described larva and pupa of U. (U.) metagana (Liu et al. 2022). Additionally, both species show sexual dimorphism in the pupal stage. These findings show that further work on immature stages of tenebrionid would be very useful to allow the possibility of species delimitation and identification in tenebrionid also on the basis immature characters.

## Supplementary Material

XML Treatment for Uloma (Uloma) intricornicula
